# Supplementary material for: Massively parallel quantification of mutational impact on IAPP amyloid formation
Source: Nat Commun. 2026 Mar 17;17:4076. doi: 10.1038/s41467-026-70611-z (PMC13144336; doi:10.1038/s41467-026-70611-z)
Supplement: Supplementary file 1 — Supplementary Figs. [file 41467_2026_70611_MOESM1_ESM.pdf]

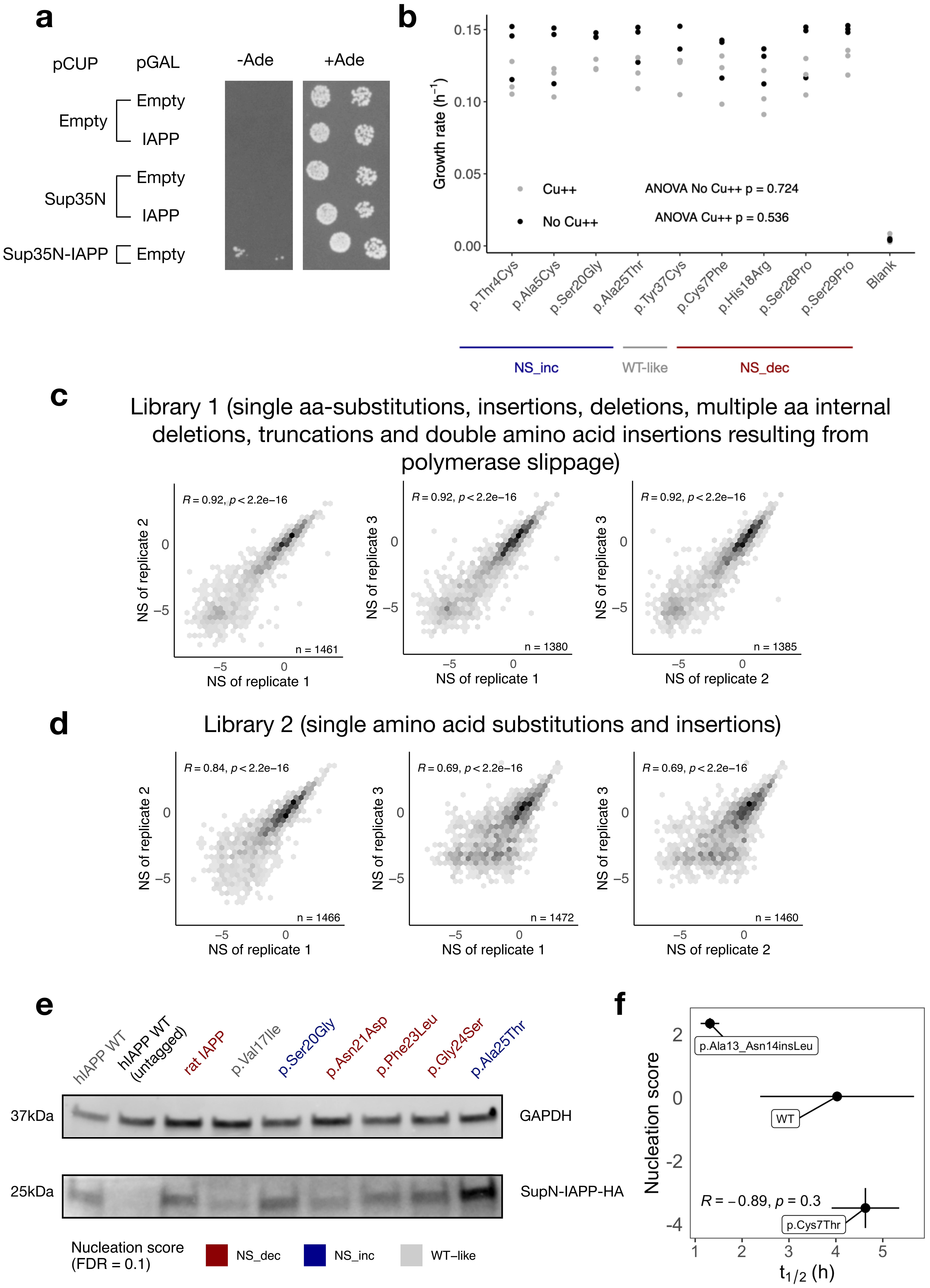
a
pCUP
pGAL
-Ade
+Ade
Empty
Empty
IAPP
Sup35N
Empty
IAPP
Sup35N-IAPP
Empty
b
Growth rate (h⁻¹)
0.15
0.10
0.05
0.00
Cu++
No Cu++
ANOVA No Cu++ p = 0.724
ANOVA Cu++ p = 0.536
p.Thr4Cys
p.Ala5Cys
p.Ser20Gly
p.Ala25Thr
p.Tyr37Cys
p.Cys7Phe
p.His18Arg
p.Ser28Pro
p.Ser29Pro
Blank
NS_inc
WT-like
NS_dec
c
Library 1 (single aa-substitutions, insertions, deletions, multiple aa internal deletions, truncations and double amino acid insertions resulting from polymerase slippage)
R = 0.92, p < 2.2e−16
n = 1461
NS of replicate 2
NS of replicate 1
R = 0.92, p < 2.2e−16
n = 1380
NS of replicate 3
NS of replicate 1
R = 0.92, p < 2.2e−16
n = 1385
NS of replicate 3
NS of replicate 2
d
Library 2 (single amino acid substitutions and insertions)
R = 0.84, p < 2.2e−16
n = 1466
NS of replicate 2
NS of replicate 1
R = 0.69, p < 2.2e−16
n = 1472
NS of replicate 3
NS of replicate 1
R = 0.69, p < 2.2e−16
n = 1460
NS of replicate 3
NS of replicate 2
e
hIAPP WT
hIAPP WT (untagged)
rat IAPP
p.Val17Ile
p.Ser20Gly
p.Asn21Asp
p.Phe23Leu
p.Gly24Ser
p.Ala25Thr
37kDa
GAPDH
25kDa
SupN-IAPP-HA
Nucleation score (FDR = 0.1)
NS_dec
NS_inc
WT−like
f
Nucleation score
p.Ala13_Asn14insLeu
WT
R = −0.89, p = 0.3
p.Cys7Thr
t1/2 (h)

Supplementary Figure 1. Validation and reproducibility of the IAPP nucleation assay. a. Yeast cells expressing the indicated Sup35 + IAPP constructs were grown under selective (-Ade) and non-selective conditions (+Ade). Protein expression was induced for 24 h with 100 μM Cu^{2+} and 2% galactose. Growth on -Ade plates is observed only when IAPP is fused to Sup35N, whereas co-overexpression of Sup35N and IAPP *in trans* does not induce growth, demonstrating that nucleation of endogenous Sup35 requires the Sup35N-IAPP fusion (n = 1). **b.** Individually measured growth rates for selected IAPP variants classified as increasing (NS_inc), decreasing (NS_dec), or with WT-like nucleation (FDR = 0.1), assessed under non-inducing (no Cu^{++}) and inducing (Cu^{++}) protein expression conditions (3 biological replicates per variant were performed). One-way ANOVA detected no significant differences in growth among variants under either condition. **c-d.** Correlation of nucleation scores between three biological replicates for each IAPP variant library (**c.** Library 1, **d.** Library 2). Each panel shows pairwise correlations between replicates (Rep 1 vs Rep 2, Rep 1 vs Rep 3, Rep 2 vs Rep 3) for the respective library. Pearson correlation coefficients (R, two-sided) and associated p-values, along with the number of variants plotted, are indicated on each plot. **e.** Protein extracts from induced cultures were examined by western blotting using an anti-HA antibody to detect HA-tagged Sup35N-IAPP constructs (n = 1). Variant labels are colour-coded according to their nucleation scores. An untagged cell lysate is shown as a negative control. GAPDH is included as a loading control. **f.** Correlation of aggregation half-time ($t_{1/2}$) and nucleation score for IAPP variants. $t_{1/2}$ values were derived from normalized ThT fluorescence curves for IAPP WT, p.Cys7Thr, and p.Ala13_Asn14insLeu peptides. Pearson correlation coefficients (R, two-sided) and corresponding p-values are indicated. Vertical error bars represent 95% confidence interval for the nucleation score estimates, and horizontal error bars represent ±1 standard deviation of the three independent measurements used to calculate $t_{1/2}$.

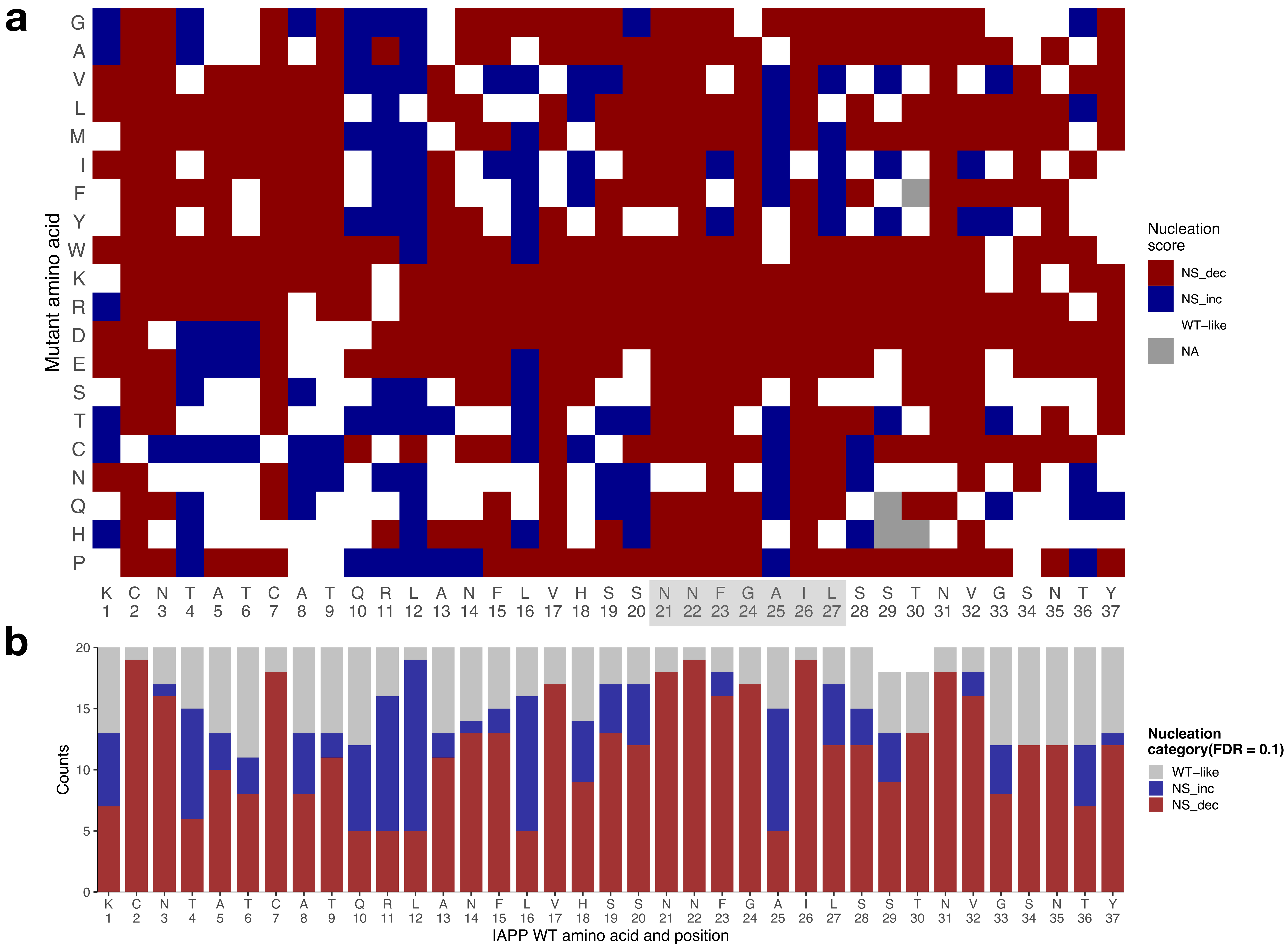

Supplementary Figure 2. Mutational effects of IAPP single amino acid substitutions. **a.** Heatmap of nucleation scores FDR = 0.1 categories for single amino acid substitutions. x-axis indicates IAPP WT position and the y-axis indicates the amino acid mutated. Variants not present in the library are represented in gray. Synonymous mutants are indicated with '*'. NNFGAIL segment is highlighted in gray. **b.** Frequency of single amino acid substitutions increasing, decreasing nucleation or having no effect in nucleation (FDR = 0.1) for each IAPP position.

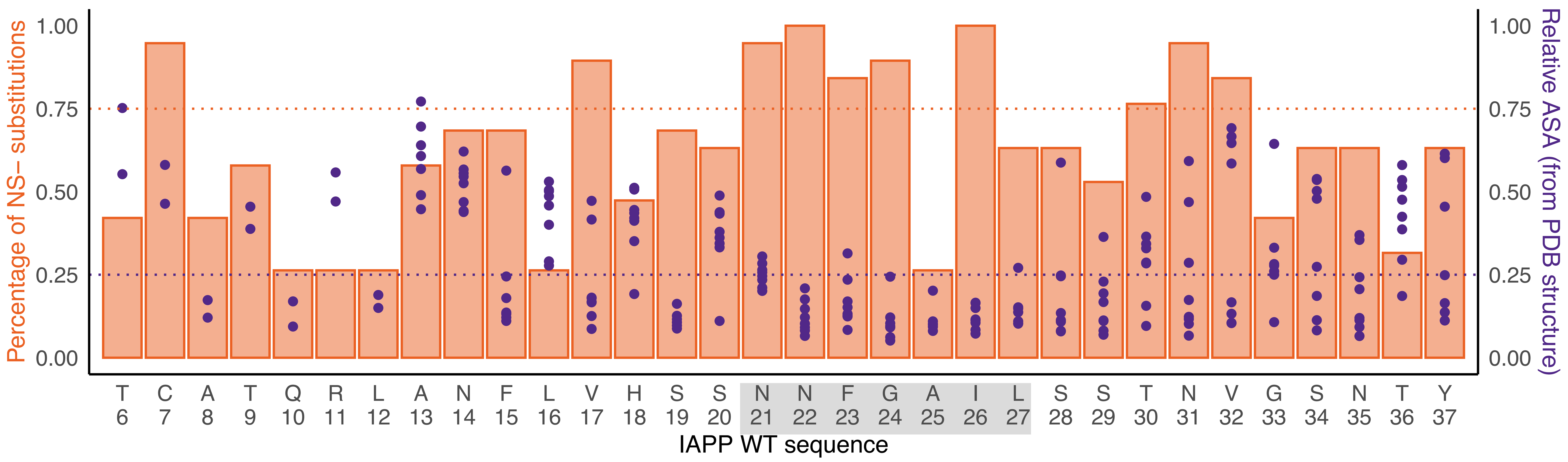

Supplementary Figure 3. Comparison of ASA and mutations decreasing nucleation per position. The bar plot and the left y-axis indicate the percentage of single amino acid substitutions that decrease nucleation (NS-, FDR = 0.1) per each IAPP residue. The orange dotted line indicates a threshold of 75% of substitutions at a residue. Purple dots and the right y-axis indicate the relative ASA per position of each of the 8 hIAPP fibril structures available from PDB. Points below the purple dotted line (relative ASA = 0.25) correspond to residues considered buried in a specific PDB structure. NNFGAIL segment is highlighted in gray.

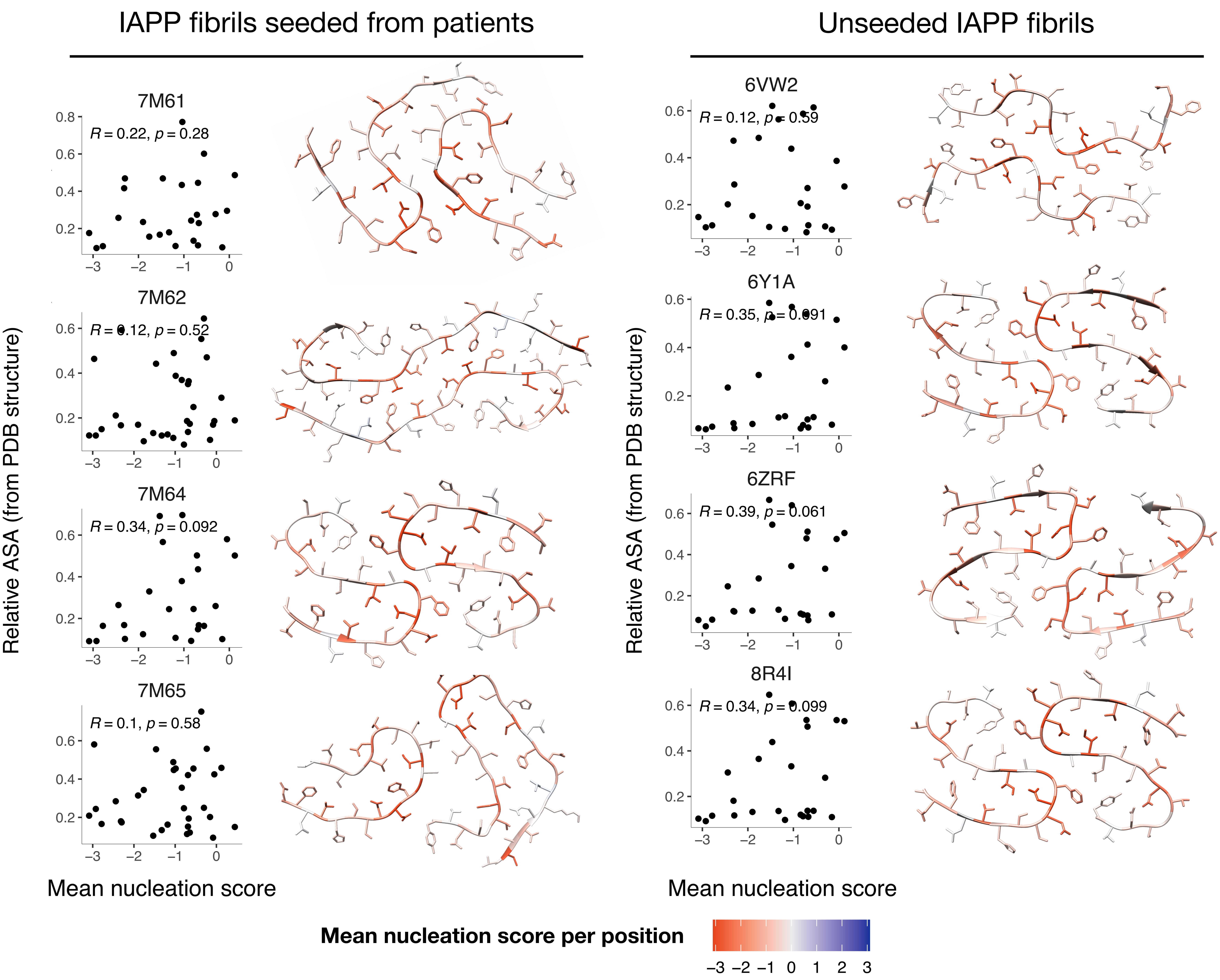

Supplementary Figure 4. Mutational effect of IAPP substitutions on all reported IAPP WT PDB fibril structures[1-5]. Correlation of mean nucleation scores of substitutions per position and available surface area extracted from all PDB reported hIAPP WT structures. Pearson correlation coefficients (R, two-sided) and corresponding p-values are indicated. hIAPP WT fibril structures are colored by the mean mutational effect of amino acid substitutions per position.

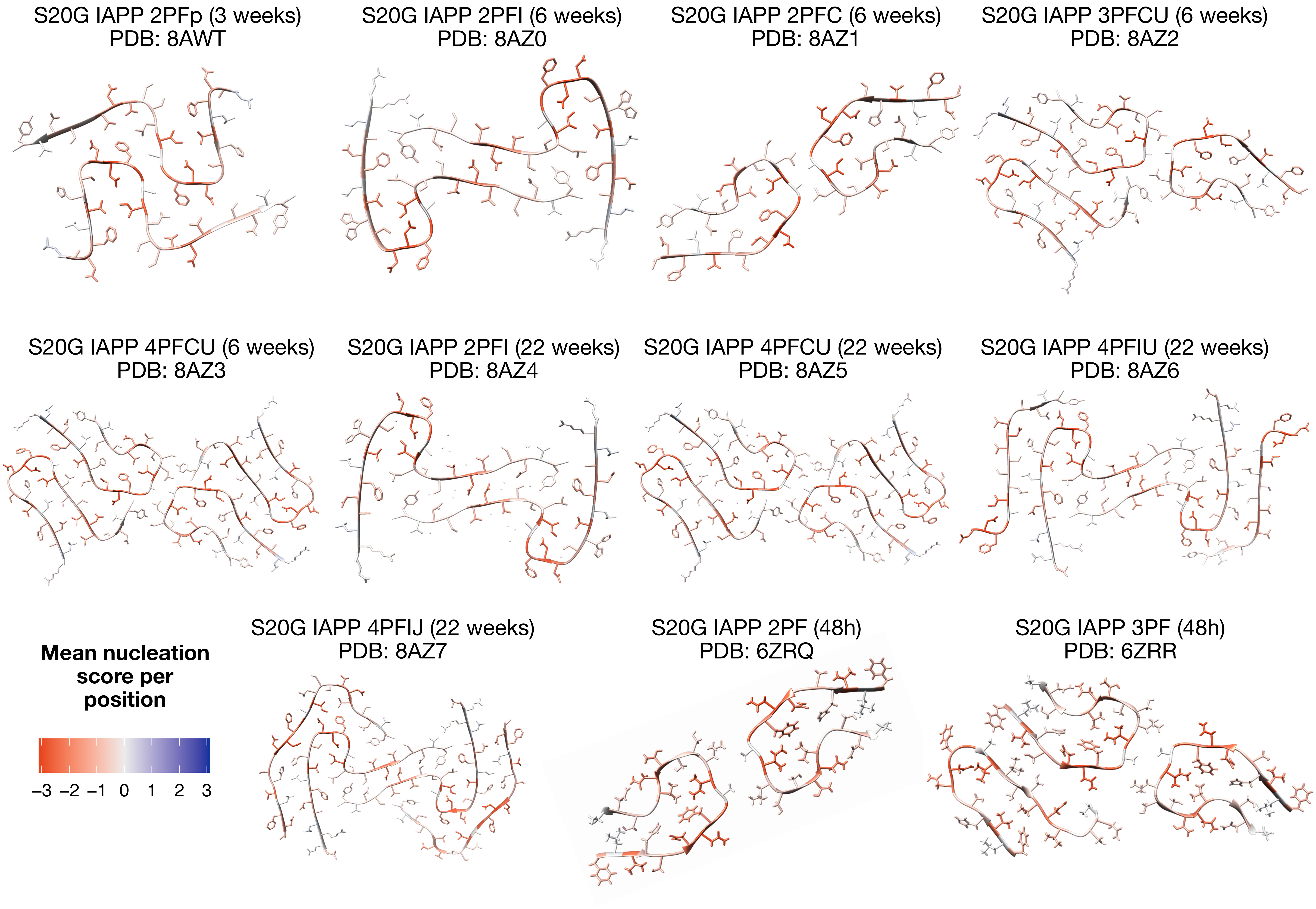

Supplementary Figure 5. Mutational effect of IAPP substitutions on all reported IAPP p.Ser20Gly PDB fibril structures[5,6]. IAPP p.Ser20Gly fibril structures from the PDB colored by the mean mutational effect of amino acid substitutions per position.

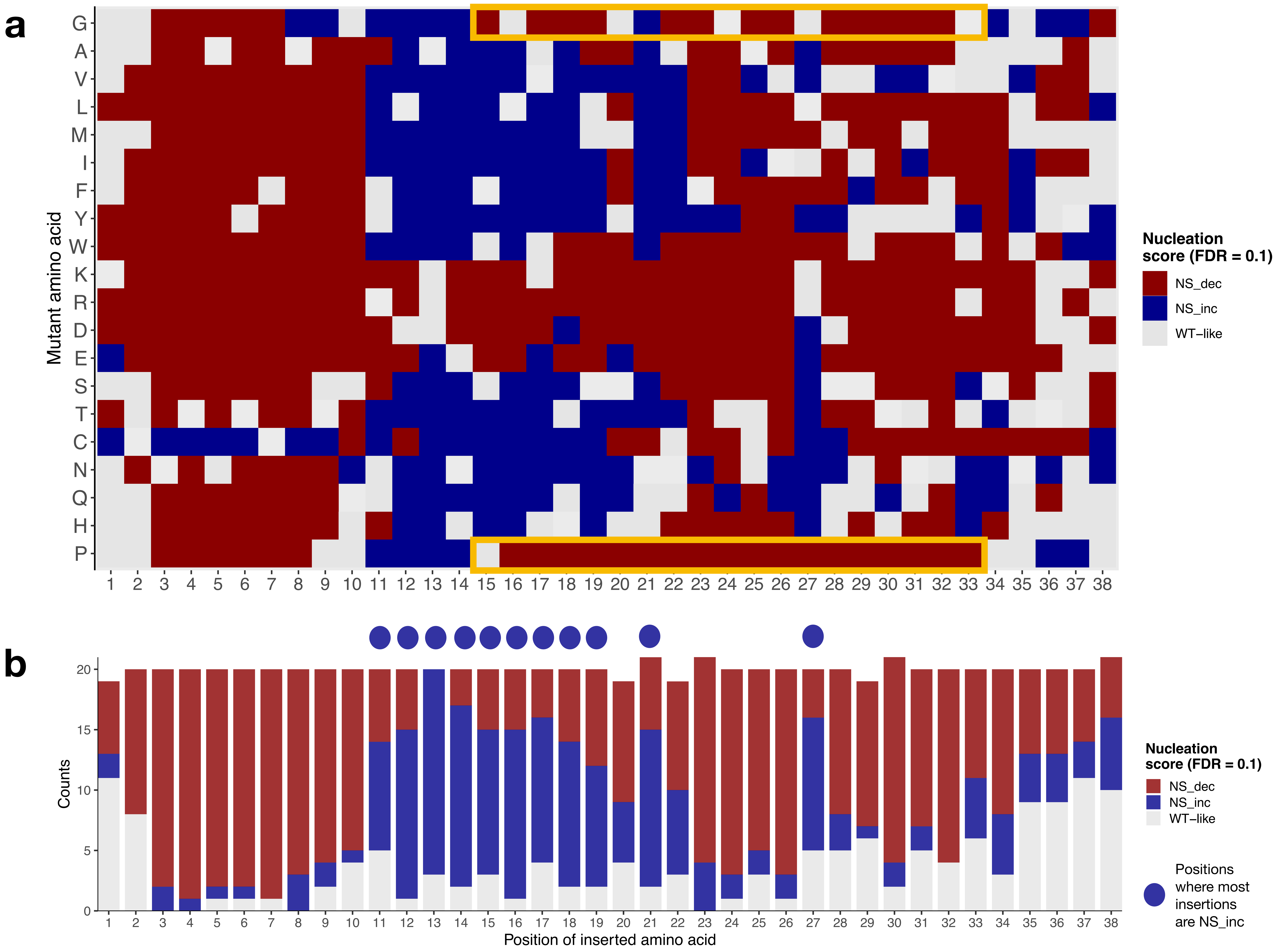

Supplementary Figure 6. Mutational effects of IAPP single amino acid insertions. a. Heatmap of nucleation scores FDR = 0.1 categories for single amino acid insertions. x-axis indicates the position of the inserted amino acid and the y-axis indicates the amino acid inserted. Variants not present are represented in gray. Yellow lines indicate a continuous stretch of 19 aa where insertions either to proline and glycine (or both) decrease nucleation. **b.** Frequency of single amino acid insertions that increase or decrease nucleation at each position. Blue circles indicate positions where NS+ mutations are the most frequent.

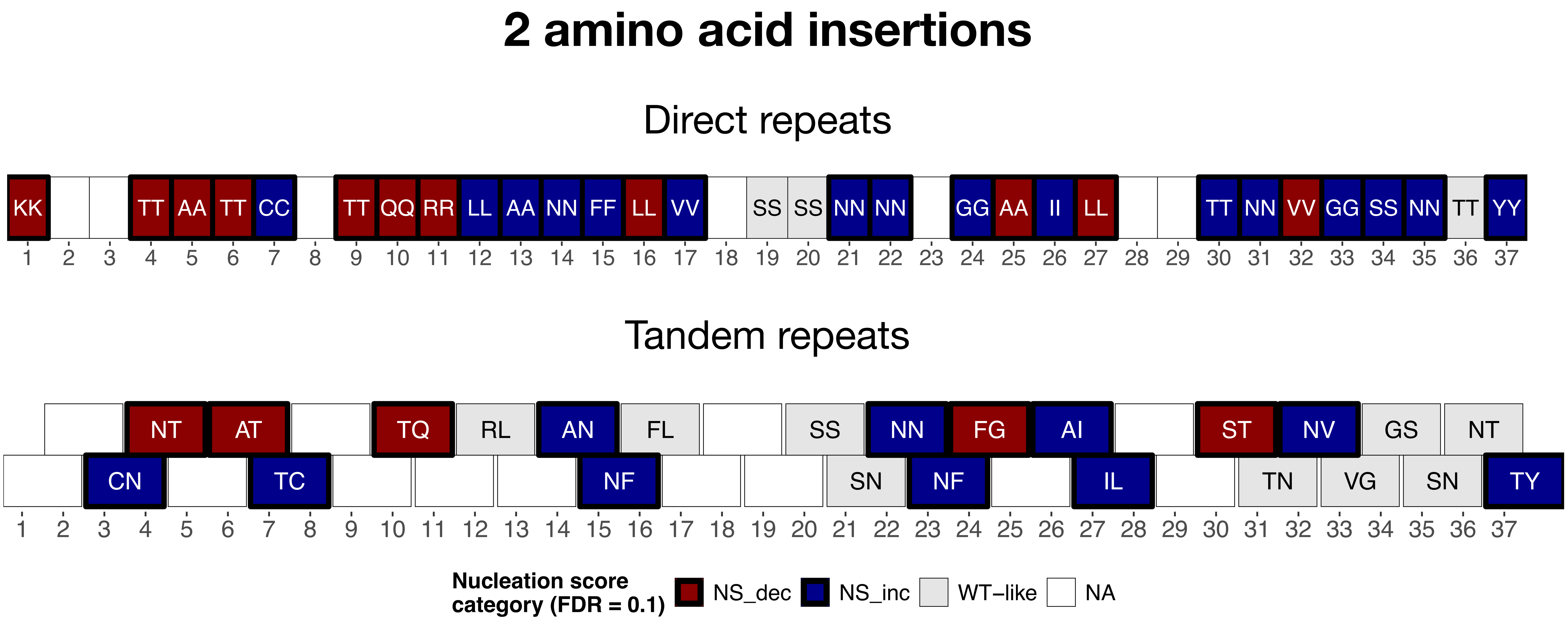

Supplementary Figure 7. Mutational effect of IAPP variants resulting from polymerase slippage. Polymerase slippage generated variants are coloured by their impact on increasing and decreasing nucleation at different FDRs. x-axis indicates the position after each pair of amino acids is inserted. The inserted amino acid pair is specified inside the squares. Variants with nucleation scores significantly different from WT (FDR = 0.1) are indicated with a wider black square.

Correlation of mutational effect of single and double amino acid insertions

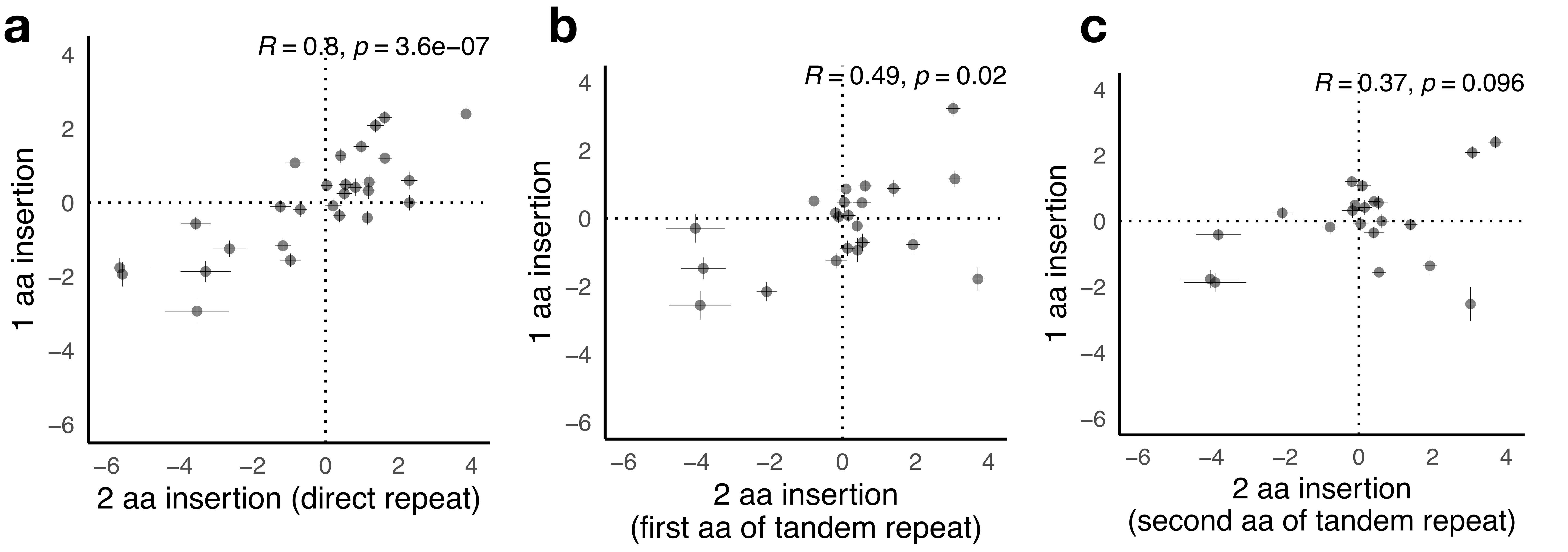

Supplementary Figure 8. Comparison between the mutational effects of single and double amino acid insertions in IAPP. a. Correlation of nucleation scores resulting from single amino acid insertions and double amino acid coming from direct repeats (same amino acid repeated twice) in the same position. **b.** Correlation of nucleation scores of single amino acid insertions with the first or the **c.** second amino acid of double amino acid insertions of tandem repeats (duplication of a pair of amino acids of the WT sequence). Pearson correlation coefficients (R, two-sided) and corresponding p-values are indicated in each plot. Vertical and horizontal error bars represent 95% confidence interval for the nucleation score estimates shown in each panel.

Correlation of mutational effect of single amino acid substitutions and insertions

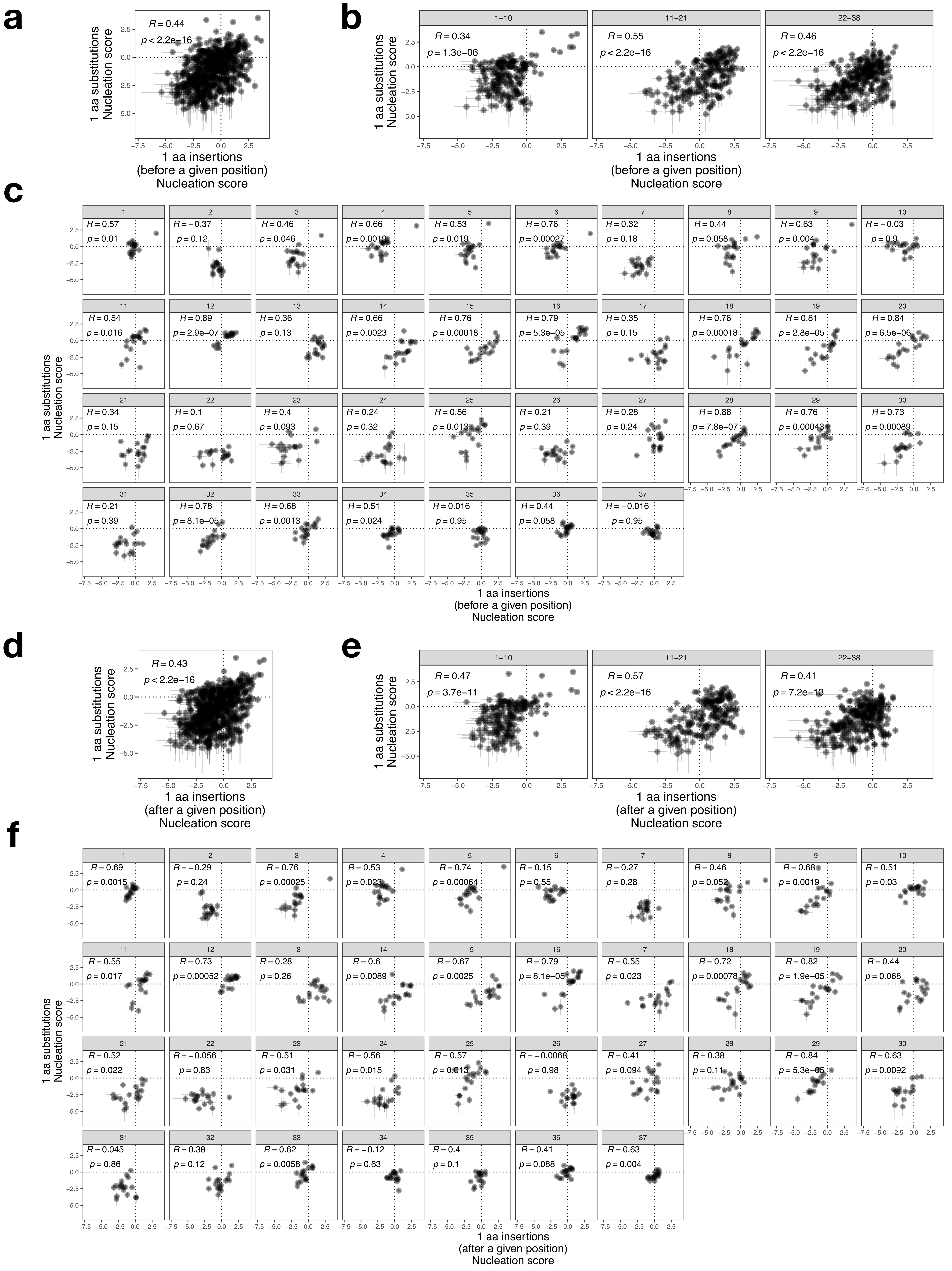

Supplementary Figure 9. Comparison between the mutational effects of insertions and substitutions in IAPP. a. Correlation of nucleation scores resulting from single amino acid substitutions and insertions of the same amino acid before the substituted position. **b.** Same as a., but values are grouped by regions defined based on mutational patterns and **c.** grouped by position. **d.** Correlation of nucleation scores of single amino acid substitutions and single amino acid insertions of the same amino acid inserted after a certain position. **e.** Correlation of nucleation scores of single amino acid substitutions and single amino acid insertions of the same amino acid inserted after a certain position grouped by regions defined based on mutational patterns and **f.** grouped by position. Pearson correlation coefficients (R, two-sided) and corresponding p-values are indicated in each plot and all panels. Vertical and horizontal error bars represent 95% confidence interval for the nucleation score estimates shown in each panel. Black squares highlight positions where correlations are significant in **c.** and **f**.

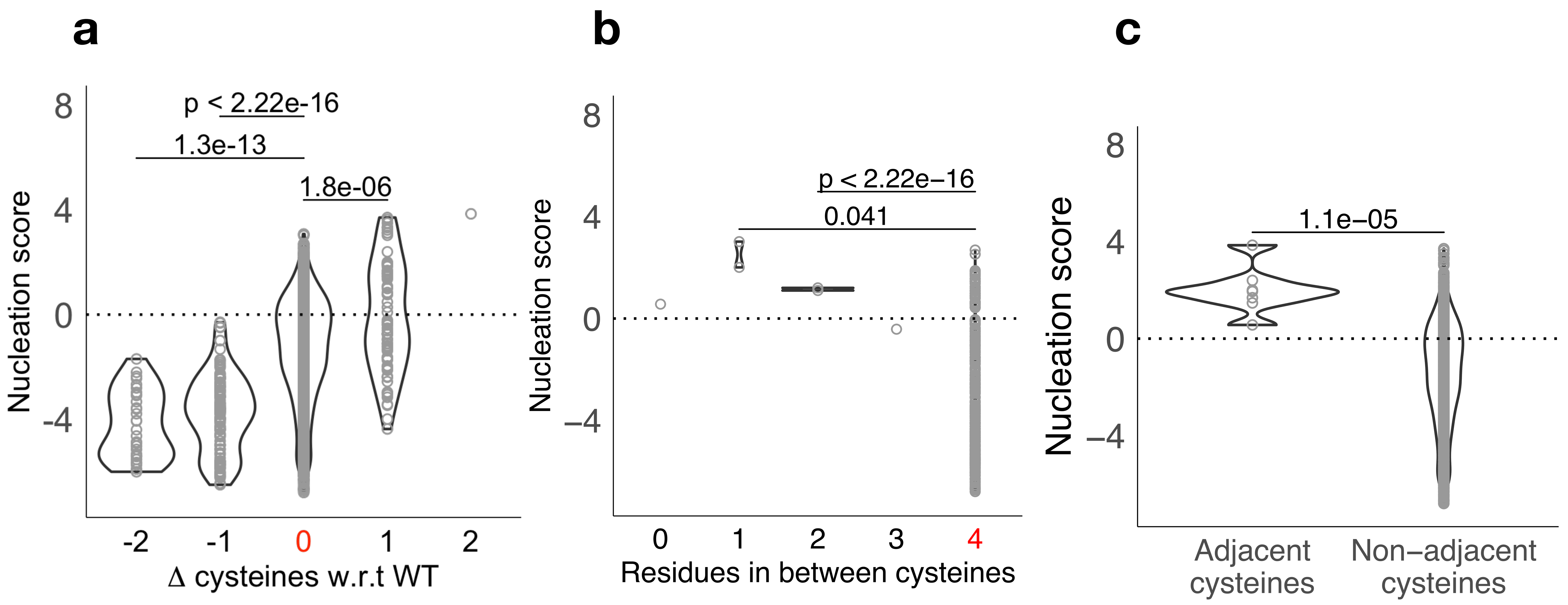

Supplementary Figure 10. Mutational effect of cysteines in IAPP nucleation. **a.** Nucleation score distributions of IAPP variants (n = 1916) grouped by the total number of cysteines in their sequence. The x-axis indicates the difference of the number of cysteine residues compared to the WT sequence (highlighted in red). **b.** Nucleation score distributions of IAPP deletion variants grouped by the sequence distance between the two cysteine residues (indicated in the x-axis). Only deletion variants containing exactly two cysteines were considered (n = 308). The corresponding cysteine-cysteine distance in the WT IAPP sequence is shown in red for reference. Nucleation score distributions of IAPP deletions grouped by the distance between the 2 cysteine residues. Only deletions containing 2 cysteines in their sequence were considered for this plot (n = 308). **c.** Nucleation scores of IAPP variants (n = 8) that have two adjacent cysteines in their sequence compared to the rest of IAPP variants. Pairwise comparisons were performed using two-sided Student's t-tests with Bonferroni correction for multiple comparison. Groups with fewer than two observations were excluded from pairwise testing due to insufficient sample size.

a

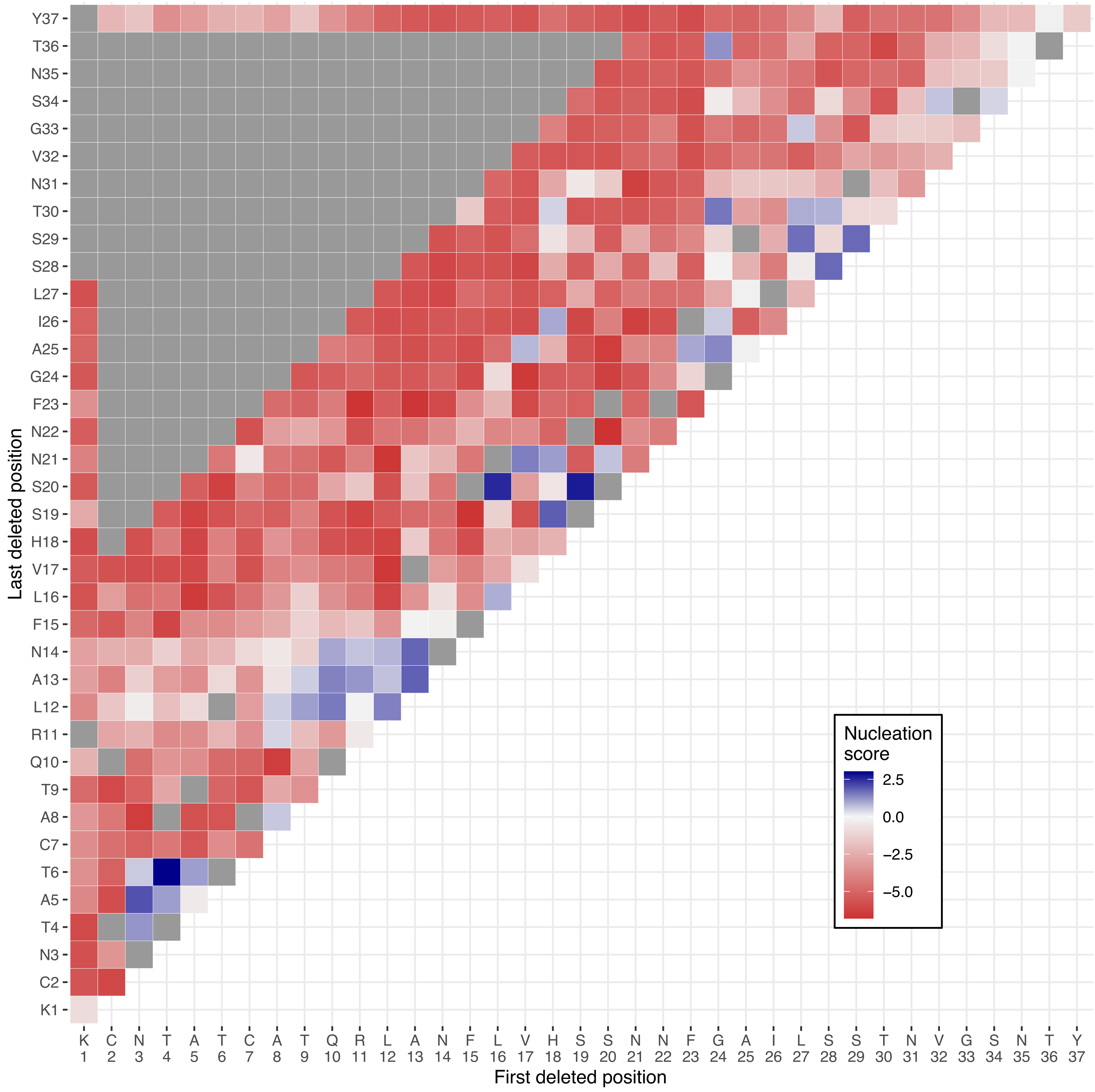

b

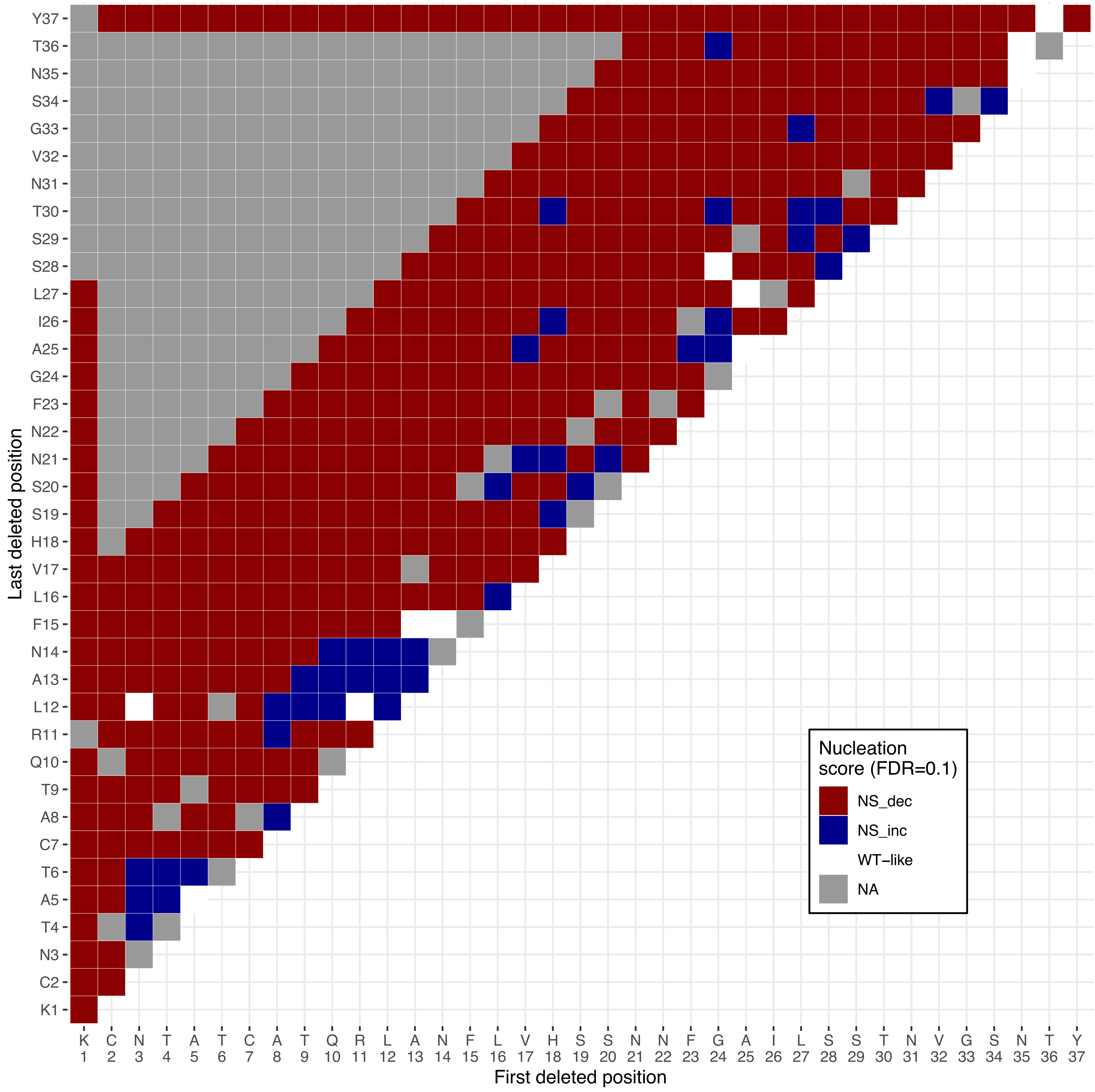

Supplementary Figure 11. Multi amino acid deletions. Heatmap of **a.** nucleation scores and **b.** nucleation score FDR categories for IAPP single and multiple amino acid deletions. x-axis and y-axis indicate the first and the last residue deleted. Missing deletions are coloured in gray.

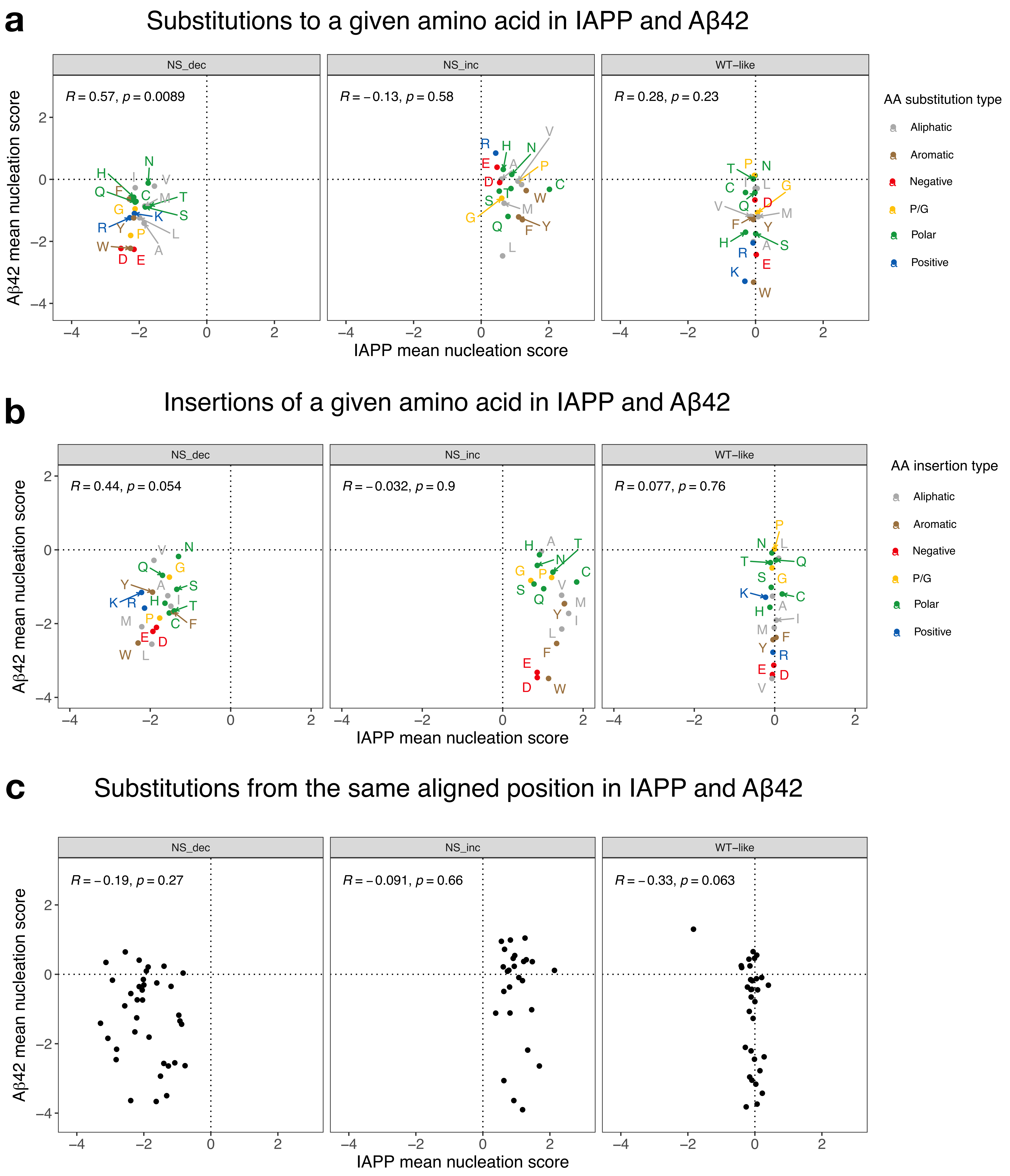

Supplementary Figure 12. Correlation of single amino-acid substitutions and insertions in IAPP and Aβ42 grouped by FDR categories. a. Correlation of mean nucleation scores for all substitutions to the same amino acid (e.g., all substitutions to valine) across the sequence in IAPP and Aβ42. Points are coloured by the class of mutant residue, with labels indicating the amino acid introduced. **b.** Same as **a.**, but for amino acid insertions, with points coloured by the class of the inserted residue and labels indicating the inserted residue. **c.** Mean nucleation scores for all mutations at the same aligned position (regardless of residue identity) are plotted pairwise for IAPP and Aβ42. In all panels, variants are separated by facets according to IAPP FDR classification. Pearson correlation coefficients (R, two-sided) and corresponding p-values are indicated.

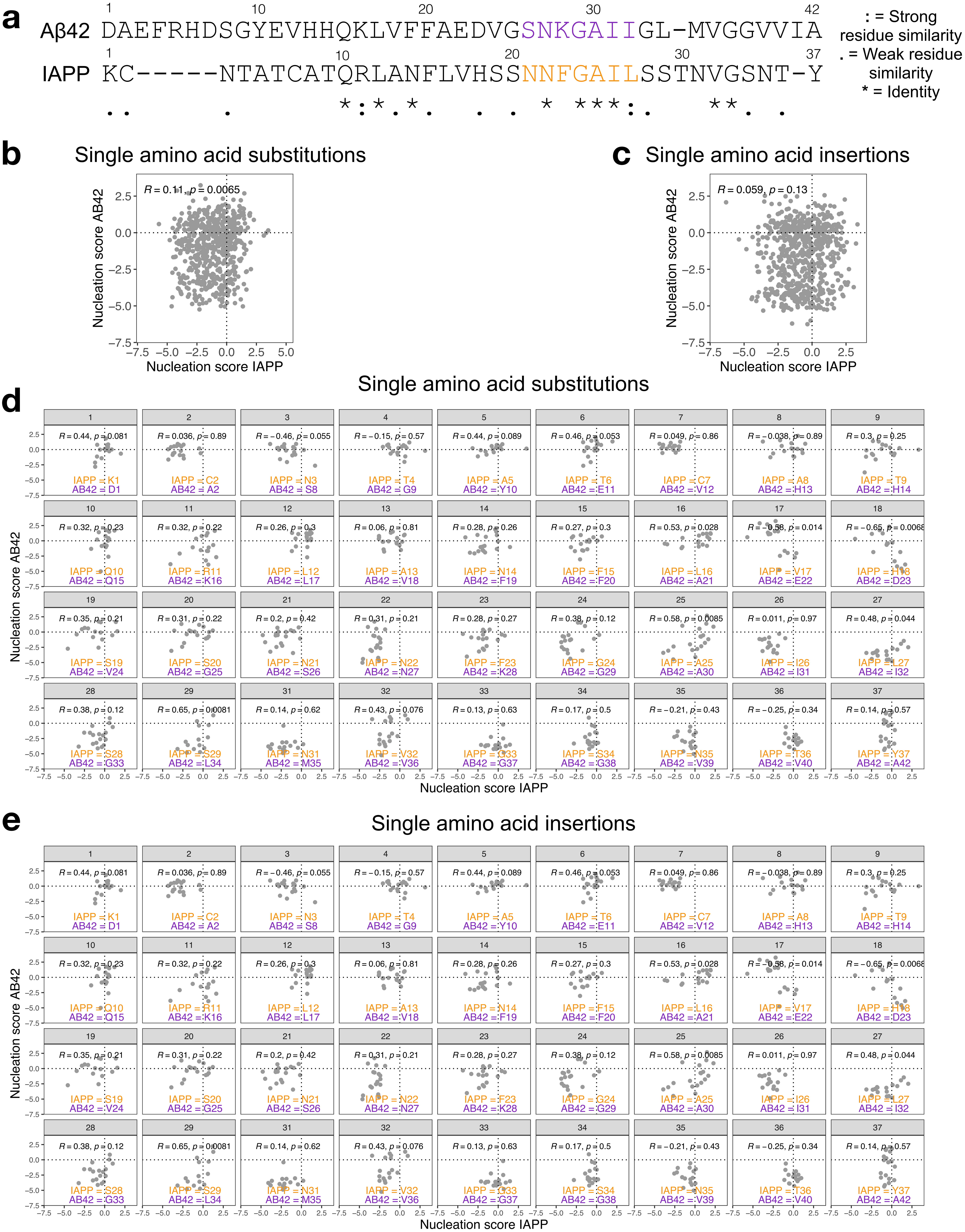
a
Aβ42 DAEFRHDSGYEVHHQKLVFFAEDVGSNKGAIIGL-MVGGVVIA
IAPP KC-----NTATCATQRLANFLVHSSNNFGAILSSTNVGSNT-Y
: = Strong residue similarity
. = Weak residue similarity
* = Identity
b
Single amino acid substitutions
R = 0.11, p = 0.0065
Nucleation score AB42
Nucleation score IAPP
c
Single amino acid insertions
R = 0.059, p = 0.13
Nucleation score AB42
Nucleation score IAPP
d
Single amino acid substitutions
Nucleation score AB42
Nucleation score IAPP
e
Single amino acid insertions
Nucleation score AB42
Nucleation score IAPP

Supplementary Figure 13. Correlation of single amino acid substitutions and insertions of IAPP and Aβ42 grouped by aligned position. a. Sequence alignment of IAPP and Aβ42 sequence by T-COFFEE[7]. WT positions of each protein are indicated above its sequence and gaps are indicated by “-”. Conservation scores are shown below the alignment: “*”, “:”, “.” indicate identical amino acids, conservative changes, and semi-conservative changes, respectively. **b.** Correlation of the nucleation scores of single amino acid substitutions and **c.** single amino acid insertions of IAPP and Aβ42 aligned by T-COFFEE. **d.** Correlation of the nucleation scores of single amino acid substitutions and **e.** single amino acid insertions of IAPP and Aβ42 grouped by T-COFFEE aligned position. WT amino acid and position are indicated in orange (IAPP) and purple (Aβ42). Pearson correlation coefficients (R, two-sided) and corresponding p-values are indicated in panels **b.**, **c.**, **d.** and **e.** Vertical and horizontal error bars represent 95% confidence interval for the nucleation score estimates shown in panels **b.**, **c.**, **d.** and **e.**

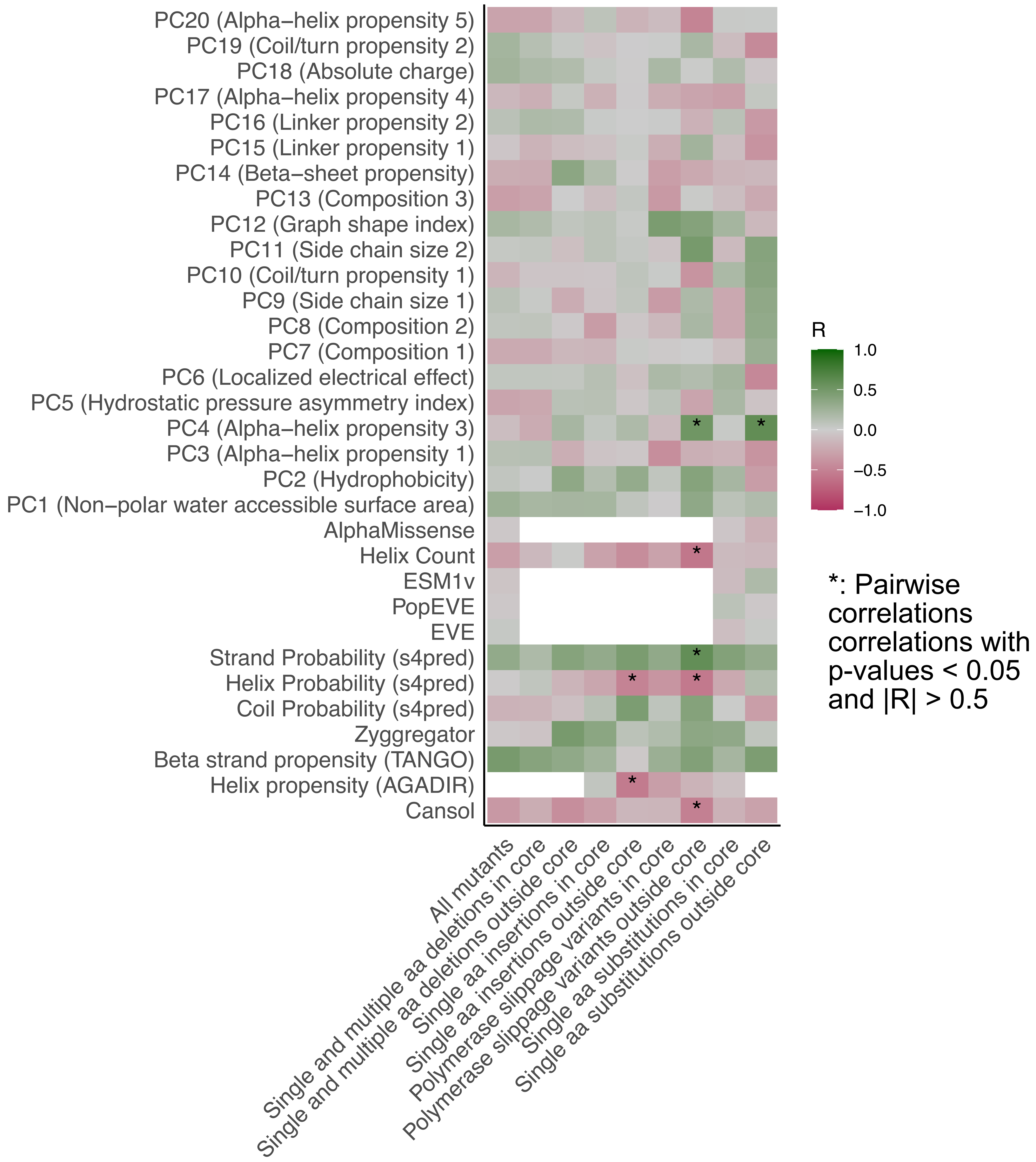

Supplementary Figure 14. Comparison of nucleation scores and aggregation and variant effect predictors. Pearson correlation of nucleation scores with the predictions of secondary structure (Tango[8], s4pred[9]), aggregation (Zyggregator[10], Camsol[11]), amino acid physicochemical properties and variant effect predictors (PopEVE[12], AlphaMissense[13]). IAPP variants are grouped by their nucleation effect or by the mutation and location type (inside core: residues 15-32), as indicated on the x-axis. Pearson correlation coefficients (R, two-sided) are shown by colour, with "*" denoting correlations with p-values < 0.05 and |R| > 0.5.

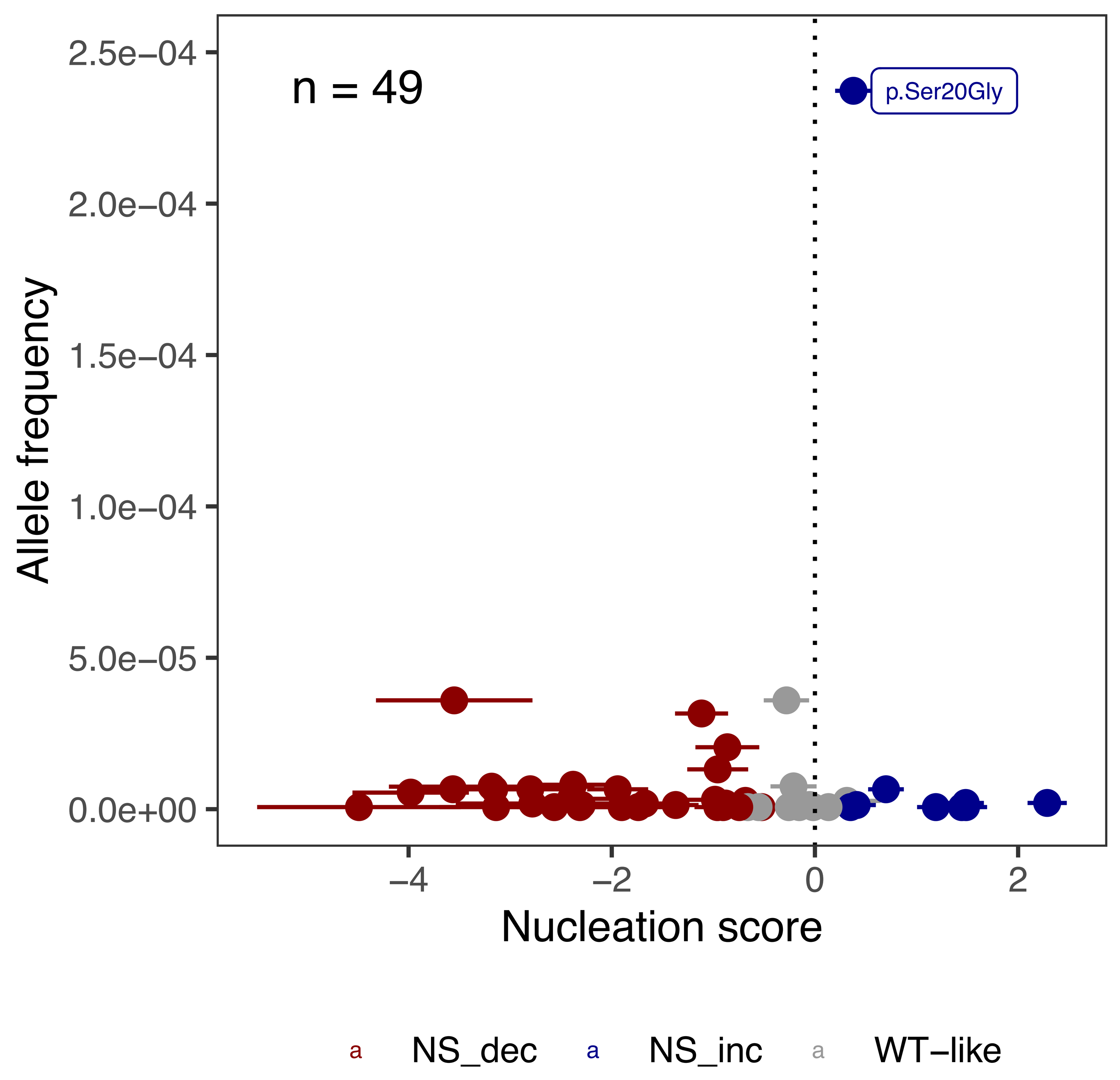

Supplementary Figure 15. Allele frequency and nucleation scores. Comparison of gnomAD[14] allele frequencies of IAPP variants and nucleation scores. Variants are colored by their FDR = 0.1 nucleation categories. Variants with allele frequency > 1e-4 are labelled. Horizontal error bars represent 95% confidence interval for the nucleation score estimates.

Supplementary References

1.Röder, C. *et al.* Cryo-EM structure of islet amyloid polypeptide fibrils reveals similarities with amyloid-β fibrils. *Nat. Struct. Mol. Biol.* **27**, 660–667 (2020).

2.Valli, D. *et al.* Improving cryo-EM grids for amyloid fibrils using interface-active solutions and spectator proteins. *Biophys. J.* **123**, 718–729 (2024).

3.Cao, Q., Boyer, D. R., Sawaya, M. R., Ge, P. & Eisenberg, D. S. Cryo-EM structure and inhibitor design of human IAPP (amylin) fibrils. *Nat. Struct. Mol. Biol.* **27**, 653–659 (2020).

4.Cao, Q. *et al.* Cryo-EM structures of hIAPP fibrils seeded by patient-extracted fibrils reveal new polymorphs and conserved fibril cores. *Nat. Struct. Mol. Biol.* **28**, 724–730 (2021).

5.Gallardo, R. *et al.* Fibril structures of diabetes-related amylin variants reveal a basis for surface-templated assembly. *Nat. Struct. Mol. Biol.* **27**, 1048–1056 (2020).

6.Wilkinson, M. *et al.* Structural evolution of fibril polymorphs during amyloid assembly. *Cell* **186**, 5798–5811.e26 (2023).

7.Notredame, C., Higgins, D. G. & Heringa, J. T-Coffee: A novel method for fast and accurate multiple sequence alignment. *J. Mol. Biol.* **302**, 205–217 (2000).

8.Fernandez-Escamilla, A.-M., Rousseau, F., Schymkowitz, J. & Serrano, L. Prediction of sequence-dependent and mutational effects on the aggregation of peptides and proteins. *Nat. Biotechnol.* **22**, 1302–1306 (2004).

9.Moffat, L. & Jones, D. T. Increasing the accuracy of single sequence prediction methods using a deep semi-supervised learning framework. *Bioinformatics* **37**, 3744–3751 (2021).

10.Tartaglia, G. G. & Vendruscolo, M. The Zyggregator method for predicting protein aggregation propensities. *Chem. Soc. Rev.* **37**, 1395–1401 (2008).

11.Sormanni, P., Aprile, F. A. & Vendruscolo, M. The CamSol method of rational design of protein mutants with enhanced solubility. *J. Mol. Biol.* **427**, 478–490 (2015).

12.Orenbuch, R., Shearer, C. A., Kollasch, A. W., Spinner, A. D., Hopf, T., van Niekerk, L., Franceschi, D., Dias, M., Frazer, J. & Marks, D. S. Proteome-wide model for human disease genetics. *Nat. Genet.* (2025) https://doi.org/10.1038/s41588-025-02400-1.

13.Cheng, J. *et al.* Accurate proteome-wide missense variant effect prediction with AlphaMissense. *Science* **381**, eadg7492 (2023).

14.Chen, S. et al. A genomic mutational constraint map using variation in 76,156 human genomes. *Nature* 625, 92-100 (2024) https://doi.org/10.1038/s41586-023-06045-0.
