# Supplementary material for: Massively parallel quantification of mutational impact on IAPP amyloid formation
Source: Nat Commun. 2026 Mar 17;17:4076. doi: 10.1038/s41467-026-70611-z (PMC13144336; doi:10.1038/s41467-026-70611-z)
Supplement: Supplementary file 2 — Description of Additional Supplementary Files [file 41467_2026_70611_MOESM2_ESM.pdf]

Description of Additional Supplementary Files

File Name: Supplementary Data 1

Description: Impact on aggregation rates for IAPP variants for which these measurements could be retrieved from the literature.

File Name: Supplementary Data 2

Description: IAPP nucleation scores obtained in this study, associated error estimates and their effect on nucleation (NS+, WT-like or NS-, FDR = 0.1).

File Name: Supplementary Data 3

Description: List of oligonucleotides used in this study.

File Name: Supplementary Data 4

Description: Burden, SKAT, Optimal Unified Tests (SKAT-O) and odds ratio for IAPP rare variant association analysis with diabetes and HbA1c levels. The different analyses were performed using either all SNPs found in the UK Biobank or by grouping them based on their effect on IAPP nucleation. Two-sided p-values and Benjamini-Hochberg adjusted p-values are noted. For the burden, SKAT and SKAT-O tests, permutation-based p-values (1000 permutations) were also computed. Odds ratios for variant groups were calculated using Firth penalized logistic regression for diabetes and linear regression for HbA1c, adjusting for age, BMI, and sex. P-values are two-sided and Benjamini-Hochberg adjusted for multiple comparisons.

File Name: Supplementary Data 5

Description: Raw variant counts, nucleation score and corresponding error estimates of Library 1 (prior to library integration).

File Name: Supplementary Data 6

Description: Raw variant counts, nucleation score and corresponding error estimates of Library 2 (prior to library integration).
