# Supplementary material for: Massively parallel quantification of mutational impact on IAPP amyloid formation
Source: Nat Commun. 2026 Mar 17;17:4076. doi: 10.1038/s41467-026-70611-z (PMC13144336; doi:10.1038/s41467-026-70611-z)
Supplement: Supplementary file 9 — Reporting Summary [file 41467_2026_70611_MOESM9_ESM.pdf]

nature portfolio

Corresponding author(s): NCOMMS-25-36335
Last updated by author(s): May 28, 2025

# Reporting Summary

Nature Portfolio wishes to improve the reproducibility of the work that we publish. This form provides structure for consistency and transparency in reporting. For further information on Nature Portfolio policies, see our Editorial Policies and the Editorial Policy Checklist.

## Statistics

For all statistical analyses, confirm that the following items are present in the figure legend, table legend, main text, or Methods section.

| n/a | Confirmed | |
|---|---|---|
| ☐ | ☒ | The exact sample size (*n*) for each experimental group/condition, given as a discrete number and unit of measurement |
| ☐ | ☒ | A statement on whether measurements were taken from distinct samples or whether the same sample was measured repeatedly |
| ☐ | ☒ | The statistical test(s) used AND whether they are one- or two-sided<br>*Only common tests should be described solely by name; describe more complex techniques in the Methods section.* |
| ☐ | ☒ | A description of all covariates tested |
| ☐ | ☒ | A description of any assumptions or corrections, such as tests of normality and adjustment for multiple comparisons |
| ☐ | ☒ | A full description of the statistical parameters including central tendency (e.g. means) or other basic estimates (e.g. regression coefficient) AND variation (e.g. standard deviation) or associated estimates of uncertainty (e.g. confidence intervals) |
| ☐ | ☒ | For null hypothesis testing, the test statistic (e.g. *F*, *t*, *r*) with confidence intervals, effect sizes, degrees of freedom and *P* value noted<br>*Give P values as exact values whenever suitable.* |
| ☒ | ☐ | For Bayesian analysis, information on the choice of priors and Markov chain Monte Carlo settings |
| ☒ | ☐ | For hierarchical and complex designs, identification of the appropriate level for tests and full reporting of outcomes |
| ☐ | ☒ | Estimates of effect sizes (e.g. Cohen's *d*, Pearson's *r*), indicating how they were calculated |

*Our web collection on statistics for biologists contains articles on many of the points above.*

## Software and code

Policy information about availability of computer code

| | |
|---|---|
| Data collection | FastQ files from high-throughput sequencing were processed with DiMSum v.1.4 using default settings (https://github.com/lehner-lab/DiMSum). Data from Seuma et al., 2022 was dowloaded from Zenodo (DOI: 10.5281/zenodo.7255570) |
| Data analysis | Github repository with the custom code to perform all the analysis and produce the figures of this work is found in: https://github.com/BEBlab/MAVE-IAPP and in Zenodo (DOI: 10.5281/zenodo.18509746)<br><br>Other software:<br>- DSSP: version 3.0.0<br>- UCSF Chimera: version 1.14.<br>- Python: version 3.8.5. |

For manuscripts utilizing custom algorithms or software that are central to the research but not yet described in published literature, software must be made available to editors and reviewers. We strongly encourage code deposition in a community repository (e.g. GitHub). See the Nature Portfolio guidelines for submitting code & software for further information.

# Data

Policy information about availability of data

All manuscripts must include a data availability statement. This statement should provide the following information, where applicable:

- Accession codes, unique identifiers, or web links for publicly available datasets
- A description of any restrictions on data availability
- For clinical datasets or third party data, please ensure that the statement adheres to our policy

Raw sequencing data are deposited in the European Nucleotide Archive under the number (ENA) under project accession number: PRJEB104038 (https://www.ebi.ac.uk/ena/browser/view/PRJEB104038). The processed data (nucleation score estimates and associated error terms) are provided in Supplementary Data 2 and have also been deposited in MaveDB. The processed dataset can be found by searching IAPP from the MaveDB landing page (https://www.mavedb.org/). The Universal Resource Name (URN) for the dataset is urn:mavedb:00001253-a.
Data retrieved from the literature and data obtained from all the experiments described in the manuscript are provided as Source Data and can be also found in Zenodo (DOI: 10.5281/zenodo.18509746).
Amyloid beta 42 dataset used for IAPP and AB comparison can be found in Zenodo (DOI: 10.5281/zenodo.7255570).
The coordinates for the PDB structures used in the study were obtained with accession: 7M61, 7M62, 7M64, 7M65, 6Y1A, 6ZRF, 8R4I, 6VW2, 8AWT, 8AZ0, 8AZ1, 8AZ2, 8AZ3, 8AZ4, 8AZ5, 8AZ6, 8AZ7, 6ZRR, 6ZRQ, 7Q4M. UK Biobank data has been accessed under the Application Number 441451 and in accordance with the UK Biobank Ethics and Governance Framework. UK Biobank (UKBB) data used in this analysis is subject to data use restrictions. Variant frequency data were obtained from gnomAD v4.1.0 (ENST00000240652.8).

# Research involving human participants, their data, or biological material

Policy information about studies with human participants or human data. See also policy information about sex, gender (identity/presentation), and sexual orientation and race, ethnicity and racism.

| | |
|---|---|
| Reporting on sex and gender | We included sex as a covariate in our analysis of the UK BioBank data. |
| Reporting on race, ethnicity, or other socially relevant groupings | No exclusion criteria were applied based on race, ethnicity, or other socially relevant groupings in our analysis of the UK BioBank data. |
| Population characteristics | The UK Biobank database comprises individuals aged 40–69 years recruited across the United Kingdom in 2006-2010. |
| Recruitment | Recruitment procedure is specified in the UK Biobank website. |
| Ethics oversight | UK Biobank has approval from the North West - Haydock Research Ethics Committee (REC reference: 21/NW/0157). All UK Biobank study participants gave written informed consent. |

Note that full information on the approval of the study protocol must also be provided in the manuscript.

# Field-specific reporting

Please select the one below that is the best fit for your research. If you are not sure, read the appropriate sections before making your selection.

☒ Life sciences ☐ Behavioural & social sciences ☐ Ecological, evolutionary & environmental sciences

For a reference copy of the document with all sections, see nature.com/documents/nr-reporting-summary-flat.pdf

# Life sciences study design

All studies must disclose on these points even when the disclosure is negative.

| | |
|---|---|
| Sample size | Sample sizes in the library cloning in both bacteria and yeast transformations were selected to represent each variant in the mutational library 50 times, to ensure losing as few as possible amino acid variants during the experiments. |
| Data exclusions | Sequencing reads that did not pass the QC filters using DiMSum v. 1.4. were excluded. For all samples, variants not designed in the library were further filtered with a custom script to retain only the designed variants. |
| Replication | 3 biological replicates per library. All attempts at replication were successful. |
| Randomization | NA. Libraries of IAPP variants are measured in parallel in the same experiment, which effectively is blind and random. The calculations are automated by code and done blindly. |
| Blinding | NA. Libraries of IAPP variants are measured in parallel in the same experiment, which effectively is blind and random. The calculations are automated by code and done blindly. |

# Reporting for specific materials, systems and methods

We require information from authors about some types of materials, experimental systems and methods used in many studies. Here, indicate whether each material, system or method listed is relevant to your study. If you are not sure if a list item applies to your research, read the appropriate section before selecting a response.

## Materials & experimental systems

| n/a | Involved in the study |
| --- | --- |
| ☐ | ☒ Antibodies |
| ☐ | ☒ Eukaryotic cell lines |
| ☒ | ☐ Palaeontology and archaeology |
| ☒ | ☐ Animals and other organisms |
| ☒ | ☐ Clinical data |
| ☒ | ☐ Dual use research of concern |
| ☒ | ☐ Plants |

## Methods

| n/a | Involved in the study |
| --- | --- |
| ☒ | ☐ ChIP-seq |
| ☒ | ☐ Flow cytometry |
| ☒ | ☐ MRI-based neuroimaging |

## Antibodies

| | |
| --- | --- |
| Antibodies used | Primary antibodies: anti-HA (ChIP grade, AB9110, Abcam; 1:1000 1% (w/v) nonfat dry milk in PBS) and anti-GAPDH (loading control, AB9484, Abcam; 1:5000 diluted in 1% (w/v) nonfat dry milk in PBS).<br>Secondary antibodies: anti-rabbit IgG-HRP (GENA934, Cytiva, 1:5000) and anti-mouse IgG-HRP, sc-516102, Santa Cruz Biotechnology, 1:5000). |
| Validation | All the antibodies used in this study were validated by the manufacturers or publications showed in the product sheets. |

## Eukaryotic cell lines

Policy information about cell lines and Sex and Gender in Research

| | |
| --- | --- |
| Cell line source(s) | Saccharomyces cerevisiae [psi-][pin-] (MATa ade1-14 his3 leu2-3,112 lys2 trp1 ura3-52) |
| Authentication | The cell line was not authenticated |
| Mycoplasma contamination | Not tested for Mycoplasma (not-applicable) |
| Commonly misidentified lines<br>(See ICLAC register) | NA |

## Plants

| | |
| --- | --- |
| Seed stocks | *Report on the source of all seed stocks or other plant material used. If applicable, state the seed stock centre and catalogue number. If plant specimens were collected from the field, describe the collection location, date and sampling procedures.* |
| Novel plant genotypes | *Describe the methods by which all novel plant genotypes were produced. This includes those generated by transgenic approaches, gene editing, chemical/radiation-based mutagenesis and hybridization. For transgenic lines, describe the transformation method, the number of independent lines analyzed and the generation upon which experiments were performed. For gene-edited lines, describe the editor used, the endogenous sequence targeted for editing, the targeting guide RNA sequence (if applicable) and how the editor was applied.* |
| Authentication | *Describe any authentication procedures for each seed stock used or novel genotype generated. Describe any experiments used to assess the effect of a mutation and, where applicable, how potential secondary effects (e.g. second site T-DNA insertions, mosiacism, off-target gene editing) were examined.* |
